# Supplementary material for: Magnitude of telemedicine utilization and associated factors among health professionals working at selected public hospitals in Southern Ethiopia
Source: PLoS One. 2025 Jan 3;20(1):e0311956. doi: 10.1371/journal.pone.0311956 (PMC11698327; doi:10.1371/journal.pone.0311956)
Supplement: S1 Checklist — (DOCX) [file pone.0311956.s001.docx]

| STROBE Statement—Checklist of items that should be included in reports of ***cross-sectional studies*** | | | |
| --- | --- | --- | --- |
|  | Item No | Recommendation | Remarks |
| **Title and abstract** | 1 | (*a*) Indicate the study’s design with a commonly used term in the title or the abstract | “Title page” |
|  |  | (*b*) Provide in the abstract an informative and balanced summary of what was done and what was found | “Abstract, page number 1” |
| Introduction | | |  |
| Background/rationale | 2 | Explain the scientific background and rationale for the investigation being reported | “Introduction, paragraph 3” |
| Objectives | 3 | State specific objectives, including any prespecified hypotheses | “Introduction, paragraph 4” |
| Methods | | |  |
| Study design | 4 | Present key elements of study design early in the paper | “Methods, paragraph 1 first line” |
| Setting | 5 | Describe the setting, locations, and relevant dates, including periods of recruitment, exposure, follow-up, and data collection | “Methods, paragraph 1” |
| Participants | 6 | (*a*) Give the eligibility criteria, and the sources and methods of selection of participants | “Methods, Population, sample size determination, and sampling procedure sub-section” |
| Variables | 7 | Clearly define all outcomes, exposures, predictors, potential confounders, and effect modifiers. Give diagnostic criteria, if applicable | “Methods, Study variables and operational definitions sub-section” |
| Data sources/ measurement | 8* | For each variable of interest, give sources of data and details of methods of assessment (measurement). Describe comparability of assessment methods if there is more than one group | “Methods, data collection tools and quality assurance sub-section” |
| Bias | 9 | Describe any efforts to address potential sources of bias | “Methods, data collection and quality assurance section paragraph 1” |
| Study size | 10 | Explain how the study size was arrived at | “Methods, Population, sample size determination, and sampling procedure sub-section” |
| Quantitative variables | 11 | Explain how quantitative variables were handled in the analyses. If applicable, describe which groupings were chosen and why | “Methods, Data processing and analysis section line 3” |
| Statistical methods | 12 | (*a*) Describe all statistical methods, including those used to control for confounding | “Methods,Data processing and analysis” |
|  |  | (*b*) Describe any methods used to examine subgroups and interactions | “Methods,Data processing and analysis” |
|  |  | (*c*) Explain how missing data were addressed | “N/A”. |
|  |  | (*d*) If applicable, describe analytical methods taking account of sampling strategy | “Methods,Data processing and analysis” |
|  |  | (*e*) Describe any sensitivity analyses | “N/A”. |
| Results | | |  |
| Participants | 13* | (a) Report numbers of individuals at each stage of study—eg numbers potentially eligible, examined for eligibility, confirmed eligible, included in the study, completing follow-up, and analysed | “Results, paragraph 1 line 1” |
|  |  | (b) Give reasons for non-participation at each stage | “N/A” |
|  |  | (c) Consider use of a flow diagram | “N/A”. |
| Descriptive data | 14* | (a) Give characteristics of study participants (eg demographic, clinical, social) and information on exposures and potential confounders | “Results, Table 2 and Figure 1” |
|  |  | (b) Indicate number of participants with missing data for each variable of interest | “N/A”. |
| Outcome data | 15* | Report numbers of outcome events or summary measures | “Results, Knowledge of respondents, Attitude, and Practice of telemedicine” |
| Main results | 16 | (*a*) Give unadjusted estimates and, if applicable, confounder-adjusted estimates and their precision (eg, 95% confidence interval). Make clear which confounders were adjusted for and why they were included | “Factors associated with Knowledge Table 4,  “Factors associated with Attitude Table 5, and factors associated with practice Table 6 |
|  |  | (*b*) Report category boundaries when continuous variables were categorized | Table 4, 5, and 6” |
|  |  | (*c*) If relevant, consider translating estimates of relative risk into absolute risk for a meaningful time period | “N/A”. |
| Other analyses | 17 | Report other analyses done—eg analyses of subgroups and interactions, and sensitivity analyses | “N/A”. |
| Discussion | | |  |
| Key results | 18 | Summarise key results with reference to study objectives | “Discussion, paragraph 1” |
| Limitations | 19 | Discuss limitations of the study, taking into account sources of potential bias or imprecision. Discuss both direction and magnitude of any potential bias | “Discussion, paragraph 10” |
| Interpretation | 20 | Give a cautious overall interpretation of results considering objectives, limitations, multiplicity of analyses, results from similar studies, and other relevant evidence | “Discussion, paragraph 2-9” |
| Generalizability | 21 | Discuss the generalizability (external validity) of the study results | “Discussion, paragraph 10” |
| Other information | | |  |
| Funding | 22 | Give the source of funding and the role of the funders for the present study and, if applicable, for the original study on which the present article is based | “Declarations, funding section” |
