# Supplementary material for: Magnitude of telemedicine utilization and associated factors among health professionals working at selected public hospitals in Southern Ethiopia
Source: PLoS One. 2025 Jan 3;20(1):e0311956. doi: 10.1371/journal.pone.0311956 (PMC11698327; doi:10.1371/journal.pone.0311956)
Supplement: Attachment — Submitted filename: PONE-D-23-25592_review1.pdf [file pone.0311956.s004.pdf]

Unfortunately, neither the pages nor the lines are numbered. So for better understanding the first page will be the title page of the article (auxiliary table must be skipped) and lines will be numbered from the top of each pages, including empty.

Page	Line	Text in the article	Comments
2	12	Epi-data version 3.1	On page 6 line 22 text «Epi-data version **4.6**» is given, please, bring this into compliance
6	22	Epi-data version 4.6	On page 2 line 12 text «Epi-data version **3.1**» is given, please, bring this into compliance
6	chapter	Study variables and operational definitions	Information about variables “Knowledge” and “Attitude” is given, please, add information about variable “Practice of Telemedicine”
7	17	83.05%	On the Figure 1 is 78.13%, please, bring this into compliance
7	19	33%	On the Figure 1 is 35.63%, please, bring this into compliance
7	26	(69.0%)	On the Figure 2 is 89.9%, please, bring this into compliance
7	27	(67.3%)	On the Figure 2 is 89.2%, please, bring this into compliance
8	25	(1.09-5.82))	1.00 in the table, please, bring this into compliance
	Fig.2	3D graph	It’s not necessary to use 3 dimensional picture to show two variables, be better if you use 2dimensional picture.
	Fig.2	Legend or “Do you know the types or classification of telemedicine?”	It's possible the colors are mixed up, please, bring this into compliance
14	7	Triangulate	Could you, please, explain this

Some grammatical notes

Page	Line	Text in the article	Comments
8	22	**After controlling the potential confounding variables by a multivariable analysis;** Age≥36 years (AOR 2.99, 95%CI=1.18-7.60),being medical doctor (AOR 3.91 95% CI=1.15-13.25), Having good knowledge (AOR 2.75 95%CI (1.54-4.89)), Presence of information sharing culture (AOR 3.95, 95%CI (1.16-13.45)), presence of practicing platform (AOR 3.01, 95%CI (1.06-8.53)) and presence of government commitment (AOR 2.52, 95%CI (1.09-5.82)) **were found to be significantly associated with telemedicine service utilization**	**After controlling the potential confounding variables by a multivariable analysis the following were found to be significantly associated with telemedicine service utilization:** age≥36 years (AOR 2.99, 95%CI=1.18-7.60),being medical doctor (AOR 3.91 95% CI=1.15-13.25), Having good knowledge (AOR 2.75 95%CI (1.54-4.89)), Presence of information sharing culture (AOR 3.95, 95%CI (1.16-13.45)), presence of practicing platform (AOR 3.01, 95%CI (1.06-8.53)) and presence of government commitment (AOR 2.52, 95%CI (1.09-5.82)).
9	12	counter parts	counteparts

13	15	counter parts	counterparts
10	1	governmentcommitment	government commitment
9	11	governmentalcommitted	governmental committed
9	2	Health professionals who are age ≥36 years were nearly 3 times more likely of utilizing telemedicine services than those health professions with younger of age groupof age	Health professionals who are age ≥36 years were nearly 3 times more likely of utilizing telemedicine services than those health professions **from** younger of age group (of age <25 years).
8	10	mmore	more
5	28	scale	scales
11	16	provider	Specialist, person
11	18	provider	Specialist, person
12	14	providers	Specialist, person

Some technical notes

**Page**	**Line**	**Text in the article**	**Comments**
3	8	Telehealth[2,3].Telehealth	space
3	18	involvedthe	space
3	26	long-termas	space
3	29	effectivenessduring	space
3	30	recognized[10,11, 13].The	space
4	1	[11,12].Different	space
4	5	[18].In	space
4	6	[5].Although	space
5	11	407.A	space
5	19	techniquesand	space
7	12	profession,nearly	space
9	2	groupof	space
9	18	30-39.6%],54.1%	space
9	19	[95%CI: 21.6-30.2%]of	space
10	6	andBangladesh	space
10	8	a [23],and	space
10	16	[24],Karachi,	space
10	16	[14].However	space
13	14	governmentalcommitted	space
13	15	hospitalshave	space
14	1	knowledgeregarding	space
8	16	respondentused	space
9	10	(1.54-4.89)].On	space
3	28	patients[6].and	dot
4	11	[20]. were	dot
12	4	[18]. and Libya	dot
