# Supplementary material for: Magnitude of telemedicine utilization and associated factors among health professionals working at selected public hospitals in Southern Ethiopia
Source: PLoS One. 2025 Jan 3;20(1):e0311956. doi: 10.1371/journal.pone.0311956 (PMC11698327; doi:10.1371/journal.pone.0311956)
Supplement: Attachment — Submitted filename: Response to the Editor.docx [file pone.0311956.s003.docx]

**Dear Florie Lacorte Canales,**

I hope this letter finds you well. I am writing in response to the recent withdrawal of our manuscript **(PONE-D-23-25592)** titled ***“Telemedicine Service Utilization and associated factors among Health Professionals Working at Selected Public Hospitals in Southern Ethiopia, 2021: A Facility Based Cross Sectional Study”.***

1. Please complete all items on the Human Participants Research Checklist that are relevant for your submission.

I have compete the form and resubmitted to the online manuscript submission system.

2. It has come to our attention that the manuscript seems to be a resubmission of the manuscript PGPH-D-22-02029, which was previously considered by the journal PLOS Global Public Health.

It is true that the manuscript was submitted to PLOS Global Public Health Journal with the manuscript ID [PGPH-D-22-02029] on Dec 18 2022 10:15AM and withdrawn on Feb 17 2023 6 by the decision of the editor. I would like to provide some additional context and explanation regarding the circumstances surrounding the withdrawal. The editor sent me an email stating that they were regret to inform me that the journal's executive editor faced challenges in securing an appropriate academic editor to review our manuscript. Additionally, the journal encountered difficulty in obtaining sufficient feedback from peer reviewers. Consequently, the editorial team reluctantly decided to return the manuscript without undergoing the formal review process. As a result, we were unable to receive any comments or suggestions from the chief and academic editors that would have required our response.

Thus, considering the significance and potential impact of our research findings, we believe that our manuscript aligns well with the focus and scope of PLoS One Journal. We have thoroughly assessed your journal's aims, objectives, and editorial policies and strongly believe that it would be a suitable platform to disseminate our research outcomes. Therefore, we kindly request your consideration for a new submission of the manuscript to PLoS One Journal.

We remain committed to the highest standards of scientific rigor and integrity, and we hope that our manuscript meets the criteria for publication in PLoS One Journal. We would be pleased to address any queries or concerns you may have regarding our manuscript. Thank you once again for considering our submission. We look forward to hearing from you soon.

**Sincerely,**

**Anteneh Fikrie** (Assistant Professor)
