# Supplementary material for: Magnitude of telemedicine utilization and associated factors among health professionals working at selected public hospitals in Southern Ethiopia
Source: PLoS One. 2025 Jan 3;20(1):e0311956. doi: 10.1371/journal.pone.0311956 (PMC11698327; doi:10.1371/journal.pone.0311956)
Supplement: Attachment — Submitted filename: 3.Response to Reviewers.docx [file pone.0311956.s005.docx]

**3 November, 2023**

**Dear Dr Yitagesu HA,
Academic Editor
PLOS ONE**

I am very delighted in your response to let me know that our manuscript has passed through the review stage and sent back to us so as to make a minor revision based on the comments given on our manuscript by yours well-qualified and experienced reviewers.

Therefore, I have revised the manuscript according to the reviewers' and editors’ comments. The revision process was conducted by **a point-by-point response** to each and every comment and questions mentioned by the respected reviewers and uploaded this letter as a separate file labeled **'Response to Reviewers'.** Moreover, a marked-up copy of our manuscript that highlights changes made to the original version has been uploaded as a separate file labeled **'Revised Manuscript with Track Changes'.** I have highlighted all the amendments in our re-uploaded manuscript file. Similarly, an unmarked version of our revised paper without tracked changes has been uploaded as a separate file labeled **'Manuscript'.**

**Comments by academic editor,**

1. **Please review your reference list to ensure that it is complete and correct.**
   a. **Answer: Thank you for your insights.** I have tried to correct all the references by incorporating the URL links to each of the references.
   b. On top of that, reference number 18, was cited based on its status at research square where it had not undergo a peer review process. So, Now, I have revised it after finding the published article at Digital Health. Therefore,
      i. **This reference has changed”** Assaye BT, Jemere AT, Nigatu AM. Knowledge, Awareness and Associated factors of Telemedicine Services among Health Professionals Working at Amhara Region Referral hospitals, northwest Ethiopia. Research square. com. March 17th, 2021. <https://doi.org/10.21203/rs.3.rs-334058/v2>” to the following
      ii. Assaye BT, Jemere AT, Nigatu AM. Knowledge and awareness of health professionals towards telemedicine services in Northwest, Ethiopia. Digit Health. 2022 Dec 1;8:20552076221143250. doi: 10.1177/20552076221143250. PMID: 36478985; PMCID: PMC9720804. [**https://pubmed.ncbi.nlm.nih.gov/36478985/**](https://pubmed.ncbi.nlm.nih.gov/36478985/)

Sincerely,

**AntenehFikrie** (BSc, MPH)

Corresponding author

address: [antenehfikrie3@gmail.com](mailto:antenehfikrie3@gmail.com)

Mobile: +251-922-465-129
