# Supplementary material for: Magnitude of telemedicine utilization and associated factors among health professionals working at selected public hospitals in Southern Ethiopia
Source: PLoS One. 2025 Jan 3;20(1):e0311956. doi: 10.1371/journal.pone.0311956 (PMC11698327; doi:10.1371/journal.pone.0311956)
Supplement: Attachment — Submitted filename: Comments for PONE-D-23-25592.docx [file pone.0311956.s006.docx]

I am grateful for the opportunity to review this manuscript. The topic appears to be timely, and I believe the findings from such studies could have the potential to make a significant contribution in the clinical domain of healthcare service delivery. However, I must note that the paper lacks proper articulation. I have provided the following comments in order to help improve the manuscript further.

**General comments**

- It would have been good if the draft manuscript could have line number.
- The paper needs substantial editorial and grammar issues

**Specific comments and suggestions**

1. **Title:**

- Since authors didn’t use any special study design, I suggest to remove ‘A Facility Based Cross-sectional Study’

2. **Authors list:**

- Give space between the middle and last name for the author ‘Wako GolichaWako’
- Put the sign ‘*’ to depict the first author (Anteneh Fikrie) after the supperscript1

3. **Authors Affiliation**

- For author 2 and 5: what does ‘Pharma College Hawassa Campus’ do you mean College of Pharmacy, Hawasa Compus’? What does South Ethiopia mean? If it is the name of the region, please add the country name Ethiopia after comma?
- For author 3: Could you make the initial letter of the words ‘program focal person’ to uppercase?
- For author 4: Could you make the initial letter of the words ‘airborn force level’ to uppercase?
- In general, I suggest to critical review the affiliation section

4. **Abstract part**

- In the conclusion section, how can you say ‘overall attitude of these healthcare professionals towards telemedicine was highly positive’? (As compared to what cutoff point is your finding 54.1% ‘highly positive’???)
- Your recommendation should be based on your finding? Have you assessed the user-friendliness of the platforms and obtained that it was a concern for the health professionals?

5. **Introduction part**

- Make your citation style consistent (citation 1 &2).
- Insert the citation after space (3^rd^ and 6^th^ lines)
- Better to start by intruding your area of interest ‘telemedicine’. As to me the first 10 lines are not relevant for readers.
- Needs substantial editing as there are various editorial issues such as space before citation and after full stop (Pages 8, 9, 10…..) and punctuation marks (page 10, unnecessary full stop mark after the reference [20]).
- In page 10 (5^th^ and 6^th^ lines) you stated ‘In Ethiopia Telemedicine services are increasingly ……..[5]’, however your reference is about telehealth guideline ‘*Federal Ministry of Health (FMOH). Tele health guideline practical tips.2020*’. Could you check it out again???
- In general your introduction section is not well articulated. I suggest reorganize it again using the following structure?

Background of the study (what is already known about the topic, the “gap” or what is not yet known about the topic) 🡪 importance / main motivation of the study (why it is important to learn the new information that your research adds) 🡪 properly formulate the research question and be specific on research objective 🡪 prospective contribution and 🡪finally, the introduction will finish with the "structure of the manuscript".

6. **Methods part**

Study design, setting, and period section

- In this section you missed the setting. Please add background information about the study settings (population, number of health facilities/hospitals (of which how many of them are pilot hospitals for telemedicine implementation), the number of health professionals specifically those who are expected to be involved in telemedicine implementation…..?)
- How the five hospitals were selected? And why?

Population, sample size determination, and sampling procedure section

- In this section, I suggest to remove the exclusion criteria ‘Health professionals who were seriously ill’, unless you came across individuals who were serious ill during your data collection. However, if you encountered individuals who were seriously ill it is better to report it.
- In sample size calculation you used unpublished paper for the proportion (ref: 23) why???
- You missed to introduce the very important part the sampling procedure. How do you approach the final 407 study participants?

Data collection tools techniques and quality assurance

- In this section, you missed the number of items used to measure telemedicine service utilization/practice.

7. **Result part**

- Since you didn’t operationalize telemedicine utilization, how do you come across with the overall prevalence (proportion) of the service to be 26%????
- Needs revision

8. **Discussion** **part**

- In page 16, ‘The possible explanation could be due to that the studies were conducted with smaller sample size and -------- high level professional or health tier system’, however the explanation contradicts your finding. This because according to your explanation, the current study was conducted with larger sample size compared to the other studies. If so how??
- How ‘sociodemographic characteristics and geographical distribution of the study participants’ affect the attitude of telemedicine utilization, so long as you are comparing health professionals working in hospitals???
- Since some of the references used for the explanation part not comparable, your explanations are not convincing.
- The discussion section is very shallow and needs extensive revision
