# Supplementary material for: Magnitude of telemedicine utilization and associated factors among health professionals working at selected public hospitals in Southern Ethiopia
Source: PLoS One. 2025 Jan 3;20(1):e0311956. doi: 10.1371/journal.pone.0311956 (PMC11698327; doi:10.1371/journal.pone.0311956)
Supplement: Attachment — Submitted filename: A point-by-point response to peer reviewers.docx [file pone.0311956.s007.docx]

**23 January, 2024**

**Dear respected reviewers,**

We truly believe that the constructive and critical comments and suggestions you have made to our manuscript have improved the quality of the article, thus I, on behalf of the authors would like to extend my earnest gratitude to your highest professionalism. As a result, we have updated the manuscript in light of your feedback, making detailed corrections to address each and every issue raised.

**A point-by-point response** **to peer reviewers**

**Reviewer #1: Comments**

1. Figure 2 and text "184<...>. Only 47(11.5%) of the respondent know about the types or
   185 classification of telemedicine (Figure: 2)." It means that 11,5% must be blue color, and 88,5% must be orange color.
   - **Response:** Thank you very much for such critical and very impressive comments. We have made a correction on it as per the recommendation.

**Reviewer #3: General comments**

1. It would have been good if the draft manuscript could have line number.
   - Response: Thank you. We have inserted the line numbers accordingly.
2. The paper needs substantial editorial and grammar issues
   - Response: Thank you and we have assessed and corrected the issues of the editorial.

**Specific comments and suggestions**

1. **Title:**
   a. Since authors didn’t use any special study design, I suggest to remove ‘A Facility Based Cross-sectional Study’
      - **Response: Removed.**
   b. Authors list: Give space between the middle and last name for the author ‘Wako GolichaWako’
      - **Response: Corrected**
   c. Put the sign ‘*’ to depict the first author (Anteneh Fikrie) after the supperscript1
      - **Response: Corrected**
   d. Authors Affiliation
      - For author 2 and 5: what does ‘Pharma College Hawassa Campus’ do you mean College of Pharmacy, Hawasa Compus’?
        - Pharma College is a renowned private owned Health Sciences and Business College established in Hawassa City, Ethiopia in 2004 G.C and now expanded to three different branches at different cities in Ethiopia.
   e. What does South Ethiopia mean? If it is the name of the region, please add the country name Ethiopia after comma?
      - Response: South Ethiopia is used to locate the direction of the College geographically not used to indicate the region.
   f. For author 3: Could you make the initial letter of the words ‘program focal person’ to uppercase?
      - Response: Corrected
   g. For author 4: Could you make the initial letter of the words ‘airborn force level’ to uppercase? - In general, I suggest to critical review the affiliation section
      - Response: Corrected
3. **Abstract part**
   - In the conclusion section, how can you say ‘overall attitude of these healthcare professionals towards telemedicine was highly positive’? (As compared to what cutoff point is your finding 54.1% ‘highly positive’???)
     - **Response:** Although the proportion of healthcare professionals that practice telemedicine and have strong understanding is lower, 54.1% of them had a positive opinion towards the practice.
   - Your recommendation should be based on your finding? Have you assessed the user-friendliness of the platforms and obtained that it was a concern for the health professionals?
     - **Response:** Thank you. The recommendation have refined as per the results.
4. **Introduction part**
   - Make your citation style consistent (citation 1 &2).
     - **Response: Corrected**
   - Insert the citation after space (3rd and 6th lines)
     - **Response: Corrected**
   - Better to start by intruding your area of interest ‘telemedicine’. As to me the first 10 lines are not relevant for readers.
     - **Response:** Thank you very much for your deep concern. We have strived to offer a concise introduction to the broader landscape of technological advancements. Our primary focus, as it pertains to this discussion, centers around the most essential aspect of these advancements.
   - Needs substantial editing as there are various editorial issues such as space before citation and after full stop (Pages 8, 9, 10…..) and punctuation marks (page 10, unnecessary full stop mark after the reference [20]).
     - **Response: Thank you all are corrected**
   - In page 10 (5th and 6th lines) you stated ‘In Ethiopia Telemedicine services are increasingly ……..[5]’, however your reference is about telehealth guideline ‘Federal Ministry of Health (FMOH). Tele health guideline practical tips.2020’. Could you check it out again???
     - **Response:** Thank you very much for your constructive comment. It was mistakenly written and now resolved.
5. **Background of the study** (what is already known about the topic, the “gap” or what importance / main motivation of the◊is not yet known about the topic) study (why it is important to learn the new information that your properly formulate the research question and be◊research adds) finally,◊ prospective contribution and ◊specific on research objective the introduction will finish with the "structure of the manuscript".

- **Response:** Thank you for such a constructive suggestion. We have incorporated accordingly.

6. **Methods part**

**Study design, setting, and period section**

- In **this** **section** you missed the setting.
  - Please add background information about the study settings (population, number of health facilities/hospitals (of which how many of them are pilot hospitals for telemedicine implementation), The number of health professionals specifically those who are expected to be involved in telemedicine implementation…...)How the five hospitals were selected? and why?
  - **Response.** We have described in details based on the comments.

**Population, sample size determination, and sampling procedure section**

- In this section, I suggest to remove the exclusion criteria ‘Health professionals who were seriously ill’, unless you came across individuals who were serious ill during your data collection. However, if you encountered individuals who were seriously ill it is better to report it.
  - **Response**: Thank you very much, unfortunately we were encountered such professionals , that is why we mentioned it.
- In sample size calculation you used unpublished paper for the proportion (ref: 23) why???
  - **Response**: Thank you very much for your deep and priceless critics in this regard. It is evident that unpublished studies must be carefully assessed for their merits and reliability before being taken into consideration. Keeping this in mind, we thought that the paper contains updated data that is highly relevant to our study in particular. Furthermore, it has been authored by a reputable institution and mentored by internationally renowned researchers or experts in the field. Additionally, it was conducted in one of the country’s largest and highest-level hospital setting, which leads us to consider that it provides valuable insights and reliable information.
- You missed to introduce the very important part the sampling procedure. How do you approach the final 407 study participants?
  - **Response.** Thank you. E have mentioned that we used a simple random sampling technique was employed to select the study participants

**Data collection tools techniques and quality assurance**

- In this section, you missed the number of items used to measure telemedicine service utilization/practice.
  - **Response. Thank you.** We use a “Yes “or “No” question to measure the practice. “Ever used TM services?”

7. **Result part**
   - Since you didn’t operationalize telemedicine utilization, how do you come across with the overall prevalence (proportion) of the service to be 26%????
     - **Response: We have operartionalized the “**Telemedicine utilization” as the use of telemedicine services as expected from the users at least once a week in both tele-consultation and tele-education services. As we have responded above, we measured it in terms of “Yes” an “No” question
8. **Discussion part In page 16,**
   - ‘The possible explanation could be due to that the studies were conducted with smaller sample size and -------- high level professional or health tier system’, however the explanation contradicts your finding. This because according to your explanation, the current study was conducted with larger sample size compared to the other studies. If so how??
   - **Response:** We have corrected the expansion according to the comments and highlighted.
   - How ‘sociodemographic characteristics and geographical¬ distribution of the study participants’ affect the attitude of telemedicine utilization, so long as you are comparing health professionals working in hospitals???
      Since some of the references used for the explanation part not comparable, your explanations are not convincing.¬
   - **Response:** we have corrected and explained appropriately. The discussion section is very shallow and needs extensive revision¬

**Reviewer #4:**

1. The author’s measure latent variables with simple Yes/No questions that cannot be directly observed but is estimated based on a series of observed variables. For example presence of an information sharing culture, presence of a practicing platform, and presence of government commitment.... what criterias need to met to say ''Yes'' (i.e presence of an information sharing culture, presence of a practicing platform, and presence of government commitment). Either you use established questions that have been validated in prior studies. Then you also have to weigh them together in roughly the same way that was validated before (Need to be operationalized).

- Response: Thank you very much for your comments. Of course we have been referred from the previously validated studies.

**Reviewer #5:**

1. **The title, objective and dependent variables are not in correspondence.**
   - **Response:** Thank you we have corrected and made in correspondence
2. The study population has to be operationalized or you should show the difference of utilization by cadre, if possible
   - **Response:** Thank you.
3. Serious clarification and permission of RHBs is mandatory to make the study ethically sounding, because the study was conducted in pilot area and it seems program evaluation.
   - **Response:** I sincerely appreciate your concern about the ethics. Actually, before the study was carried out, the Pharma College Institutional Review Board granted ethical permission for it. Additionally, because one of the co-authors had been employed by the Regional Health Bureau (RHB) during the study period, the RHB was aware of the research.
4. Line number 44-46 should be revised or omitted
   - **Response:** Thank you. Corrected
5. Conclusion and recommendation should comprehensive and specific to the result of findings
   - **Response:** Thank you. We corrected it
6. Line 59-60 is not as such essential
   - **Response:** Thank you. We removed it
7. Some of references were miscited that you have go through them.
   - **Response:** Thank you.
8. Line number 85-90 is not clear (negative conjunction simultaneously)
   - **Response:** Thank you very much for your significant comment. We have corrected the problems.
9. Selection criteria of health facilities have to clearly stated
   - **Response:** Thank you.
10. p-value used for sample size calculation was smaller than the result of some other articles (57.4% of the published article conducted in Northwest Ethiopia
    - **Response:** Thank you. The 57.4% was the proportion of participants who had high awareness towards telemedicine services in the specified study. However, we used a proportion for practice.
11. for which variable you calculated Cronbach's alpha? make it clear

- **Response:** Thank you. A reliability analysis of the questionnaires was checked and Cronbach’s alpha showed the questionnaire were passed the acceptable reliability number (0.975) for knowledge and (0.76) for attitude.

12. since, the response of participants is 100%, why non-response rate, was used??
    - **Response:** We were unsure if we would achieve a 100% response rate during the development of the proposal, considering the possibility of non-response bias that could be introduced during the data collection process. This bias may occur when certain individuals choose not to participate or provide incomplete responses. Therefore, to address this concern, we considered the non-response rate in order to assess if there were any significant differences between respondents and non-respondents. This analysis was conducted to ensure the validity and generalizability of the findings.
13. How could you compute the availability of practicing platform to become 78.13%? From the table of factors we find that the sum total of participants who responded yes for availability of platform were 99+230=329 that give us 329/407%=80.83**.**

- **Response:** Thank you very much for your priceless comment. It was an editorial mistake. We have corrected it.

14. in discussion section, no evidence that support the significance of the above variables with knowledge, attitude and practice/utilization of telemedicine. Please, be specific and evidence based.
    - **Response**: Thank you very much. We revised the discussion accordingly.

**Thank you!!!**
