# Supplementary material for: Magnitude of telemedicine utilization and associated factors among health professionals working at selected public hospitals in Southern Ethiopia
Source: PLoS One. 2025 Jan 3;20(1):e0311956. doi: 10.1371/journal.pone.0311956 (PMC11698327; doi:10.1371/journal.pone.0311956)
Supplement: Attachment — Submitted filename: Comments_R2.docx [file pone.0311956.s008.docx]

I am grateful for the opportunity to review this manuscript. The topic appears to be timely, and I believe the findings from such studies could have the potential to make a significant contribution in the clinical domain of healthcare service delivery. However, I must note that the paper lacks proper articulation. I have provided the following comments in order to help improve the manuscript further.

1. **Introduction part**

- Still I am not sure that you have used an appropriate citation style? What kind of citation style you have used? [1, & 2]

2. **Methods part**

- How the five hospitals (Leku Primary hospital, Shone Primary Hospital, Shinshicho Primary Hospital and Wacha Primary Hospitals) were selected? And why?

Population, sample size determination, and sampling procedure section

- In this section, I suggest to remove the exclusion criteria ‘Health professionals who were seriously ill’, unless you came across individuals who were serious ill during your data collection. However, if you encountered individuals who were seriously ill it is better to report it.
- In sample size calculation you used unpublished paper for the proportion (ref: 23) why???
- You missed to introduce the very important part the sampling procedure. How do you approach the final 407 study participants?

Data collection tools techniques and quality assurance

- In this section, you missed the number of items used to measure telemedicine service utilization/practice.

3. **Result part**

- Since you didn’t operationalize telemedicine utilization, how do you come across with the overall prevalence (proportion) of the service to be 26%????
- Needs revision

4. **Discussion** **part**

- In page 16, ‘The possible explanation could be due to that the studies were conducted with smaller sample size and -------- high level professional or health tier system’, however the explanation contradicts your finding. This because according to your explanation, the current study was conducted with larger sample size compared to the other studies. If so how??
- How ‘sociodemographic characteristics and geographical distribution of the study participants’ affect the attitude of telemedicine utilization, so long as you are comparing health professionals working in hospitals???
- Since some of the references used for the explanation part not comparable, your explanations are not convincing.
- The discussion section is very shallow and needs extensive revision
