# Supplementary material for: Magnitude of telemedicine utilization and associated factors among health professionals working at selected public hospitals in Southern Ethiopia
Source: PLoS One. 2025 Jan 3;20(1):e0311956. doi: 10.1371/journal.pone.0311956 (PMC11698327; doi:10.1371/journal.pone.0311956)
Supplement: Attachment — Submitted filename: Plos One review report.docx [file pone.0311956.s011.docx]

**Dear Plos One Publishers,**

**From:	Dr. Daniel Mengistu (PhD), Associate Professor**

**Head, Department of Nursing**

**College of Health Sciences**

**Addis Ababa University**

**Addis Ababa, Ethiopia**

**Manuscript Reviewer’s Report**

MS N0: PONE-D-23-25592R3

I would like to begin by expressing my gratitude for being assigned to the manuscript evaluation.

I acknowledge authors efforts and their consideration of the telemedicine services utilization among health professionals employed at public hospitals in Southern Ethiopia. I have summarized my review under the strong sides of the study and also of concern that need improvement to have consideration for the publication.

**Strong sides of the study**

Title:

- Timely research since we are in the information era and health professional no exception.
- Clear.

Abstract:

- Provided clear background, methods, sample size and selection, findings and conclusions.

Methods:

- Technically sounds.
- Design was appropriate to address the research question.
- Sample selection provided participants with equal chances of participating in the study.
- Statistical analyses were appropriate to data level
- The study conforms to the ethical standards
- Study limitations were acknowledged.

**Areas of concern**

| General | - The writing quality needs improvement. The entire document needs professional language editing. - The purpose focused on factors than the dependent variable. |
| --- | --- |
| Abstract | - Should provide a brief background characteristics of participants. |
| Methods  Are the methods described in sufficient detail to understand the approach used?  Are appropriate statistical tests applied? | - Authors need to state how the hospitals were selected for the study. - It is unclear how the authors excluded those who were **seriously ill** in survey study participants. - The data collection tool validity is not known. - Telemedicine utilization (the dependent variable) was not operationally defined. Hence, readers may understand it differently. - There is an item sample validity concern for the knowledge questionnaire. - How the independent variable practice measured was not clear. - Similarly, the difference between Telemedicine **Utilization** (DV), and the factor, **Practice** (as IV) is not clear. They seem one and same. If authors accept this comment, they have to remove the factor ‘Practice’ and reanalyze the data again. - In the abstract section Epidata v.3.0 use was stated but in in the analysis section Epidata v 4.6 use for data cleaning was stated. This inconsistency should be addressed. - A Cronbach’s alpha .76 was reported as reliability analysis but which questionnaire (knowledge/attitude/practice) it addressed was unclear. |
| Results: | - Analyses should be based on research question/objectives and follow pattern. - Knowledge’s finding should be summarized as per the operational definition provided and should be presented with an appropriate figure. The same works for the attitude. |
| Discussion | - Lacked focus on the primary outcome/dependent variable (telemedicine utilization) and mainly done for factors. |
| Conclusion | - While interpretations for the knowledge and practice can be considered, the conclusion for the attitude stated as **highly positive** is not acceptable and lacked data backed conclusion. |

**Reviewer’s Recommendation**

Major revision required.

Regards,
