# Supplementary material for: Magnitude of telemedicine utilization and associated factors among health professionals working at selected public hospitals in Southern Ethiopia
Source: PLoS One. 2025 Jan 3;20(1):e0311956. doi: 10.1371/journal.pone.0311956 (PMC11698327; doi:10.1371/journal.pone.0311956)
Supplement: Attachment — Submitted filename: Reviewer comments.docx [file pone.0311956.s010.docx]

**Reviewer comments:**

**on the manuscript entitled: Telemedicine Service Utilization and associated factors among Health Professionals Working at Selected Public Hospitals in Southern Ethiopia: A Facility Based Cross Sectional Study (PONE-D-23-25592_R3)**

Abstract:

Line 23: telemedicine service practice among healthcare professionals in the **pilot** public hospitals: can pilot be used for actual data collection, clarification is needed?

Introduction

1. Use either health care or healthcare throughout the manuscript.
2. World Health Organization (WHO) has defined telemedicine as ‘‘A rapid access to shared and remote medical expertise by means of telecommunication and information technologies, no matter where the patient or relevant information is located’’ [6]. Line 58-61. Why used the quotation “-------“??
3. I believe there are some publishable elements in this paper but I believe the structure and scope of this manuscript need reworking. Relevant policy implications needed to be addressed.

Methods

1. the capital of **Sideman** region in line 101: Is the author want to say Sidama region?
2. Line 113-114: Healthcare Services 114 Provides: Women's Health, Obstetrics and Gynecology, Pediatrics, and Surgery; I believe that this belongs to the Introduction section.
3. Line 138: Data collection tools techniques: Tools and techniques seems synonym? I believe the 'data quality assurance’ sections are not required. You can just mention that these personnel were given training in the relevant paragraph without having a separate section in the manuscript.
4. Line 139: The data were collected using self-administered questionnaire. What about the reliability and validity of the questionnaire? From which of the standard type of questionnaire was the study instrument developed are needed to address??
5. Lines 142-161: The knowledge level about telemedicine service was assessed using “Yes’ or “No” questions. Four items were used to assess the participants’ level of telemedicine knowledge. Each item was assessed with a 2-point scales having a score of (0=No, 1=Yes). The overall score could range from a minimum of 0 to a maximum of 4. Twenty three five point Likert scale was used to assess attitude (5 = Strongly Agree, 4 = agree, 3 = neutral, 2 = disagree and 1 = strongly disagree) related to telemedicine service. Participants were asked whether there was established culture within their organization or community that promotes the sharing of information. The question was structured as, "Is there a culture of information sharing in your organization/community? (Yes/No)". Then participants responded "Yes" if there was information sharing culture or "No" if there was not. Regarding the presence of a practicing platform; participants were asked whether there was a platform or system in place that facilitates the practice of information sharing in their institution. The question was framed as, "Is there a practicing platform for information sharing in your organization/community? (Yes/No)". Accordingly, the participants responded "Yes" if there was a dedicated platform for information sharing or "No" if a platform did not exist. On the other hand, participants were asked whether the government demonstrates commitment towards promoting information sharing. The question presented as, "Is there government commitment towards information sharing in your institution? (Yes/No)". Participants responded "Yes" if there was visible commitment from the government or "No" if there was a lack of government support in this regard.

I believe that the above section lines 142-161 should be under the heading **Measures**!

Results

1. Line 211: 341 (83.8%). And Line 212: 210(51.6%); I believe that between number and percentage spacing is not needed.
2. Line 562: the year 2022; However, Table 1. Cross-sectional study was conducted from June 1-30, 2021 among randomly selected 26 407 health professionals working at Pilot Hospitals in Southern Ethiopia mentioned in methods section??
3. The 3 Figures found in supporting information could not be found with the manuscript?

Discussion

Would the authors give implications of the study??
